# The role of age inequalities in cause of death in the slow pace of epidemiological transition in India

**DOI:** 10.1038/s41598-022-23599-7

**Published:** 2022-11-24

**Authors:** Suryakant Yadav, Arokiasamy Perianayagam, Shivani Anil Patel, Solveig Argeseanu Cunningham

**Affiliations:** 1 Department of Bio-Statistics and Epidemiology, International Institute for Population Sciences (IIPS), Mumbai, 400 088 India; 2 National Council of Applied Economic Research, New Delhi, India; 3 Rollins School of Public Health, Emory University, Atlanta, USA

**Keywords:** Epidemiology, Epidemiology, Cardiovascular diseases, Respiratory tract diseases, Asthma, Chronic obstructive pulmonary disease, Infectious diseases, HIV infections, Tuberculosis, Diseases, Health occupations, Medical research, Risk factors, Health care, Health policy, Health services, Occupational health, Public health, Quality of life

## Abstract

In developed countries, low disparity in lifespan contributed by the reduction in the burden of noncommunicable diseases (NCDs) is the key to advances in epidemiological transition. Contrarily, India passing through a phase of the dual burden of CDs and NCDs shows a heavy burden of NCDs responsible for the high disparity in lifespan. The Gini coefficient was decomposed for examining the contribution of 22 causes of death and their repercussions for inequality in age at death for 30 years between 1990–1994 and 2015–2019, using Global Burden of Disease data. The outcomes of the study reveal that India’s epidemiological transition has been just modest on account of high inequality in mortality by NCDs emplaced in the middle through old age despite a consistent mortality decline at infant through old age for communicable diseases (CDs). The structural changes in causes of death structure is shaped by CDs rather than NCDs, but overall bolstered by the adult mortality decline, especially in women. However, the process is restrained by the small contribution of the middle age group and a benign contribution of old mortality decline owing to the low threshold age. India needs to target health interventions in seeking significant mortality decline in the middle age group of 50–69 years that is warranted for epidemiological transition apace as evident in the developed nations.

## Introduction

Many developed countries in the past long-term have conformed with the course of epidemiological transition as described by Omran’s theory^1,2^. Omran’s epidemiological transition^2^ ardently advocates the substitution of communicable diseases (CDs) by the heavy burden of noncommunicable diseases (NCDs) while passing from the phase of ‘*Age of Receding Pandemics’,* and until the *‘Age of Degenerative and Man-made Diseases*.’ However, aberrations are also highlighted^3–7^, especially with the increase in the pathogenesis and aetiology of chronic diseases and also, imbricated causations across infectious and chronic diseases^8–10^ that are entwined with man-made diseases^11^. The epidemiological transition shows strong linkages with the advances in mortality transition intrigued by the causes of death structure^12,13^. Structural changes in causes of death and progression in epidemiological transition is majorly driven by the larger reduction in premature deaths at ages^14,15^ below the threshold age that separates premature deaths from old ages^16^. The same diseases show affirmative (positive) role at premature ages whereas show opposing (negative) roles in old ages^17,18^ owing to the threshold age^19^. This disparateness of affirmative and dissenting roles about the threshold age of same causes of death is conspicuous in the analysis of lifespan disparity (e^†^) or inequality in age at death^20,21^ measured by Gini coefficient at birth (G_0_)^22,23^ as compared to life expectancy at birth (e_0_). Developed countries passing through low mortality regime have shown significant developments in the causes of death structure, low inequality in age at death^24^, and mortality and/or morbidity compression^25,26^; however, these phenomena stand modest in developing countries^27^. Among these fundamental phenomena, exploration of inequality in age at death by age-sex and causes of death deciphers scrupulous assessments of the structural changes in causes of death and hence the progress of epidemiological transition.

Developed countries such as the European Unions, New Zealand, Japan, and England & Wales experience apace in epidemiological transition because of greater reduction in premature mortality, high threshold age, and swift structural changes in cause of death^28^. The USA’s health policies asserted for the reduction in burden of chronic NCDs^29^; however, the results over time exhibited higher e^†^ and lower e_0_ compared to other developed countries^30,31^. Shkolnikov et al.^23^ demonstrate higher G_0_ values in the USA than in the UK attributable to lesser reduction in premature mortality though both countries show similar threshold age, i.e. 80 years and above^32^. However, developing countries such as India and South Africa show the threshold age of 71.3 years and 58.7 years, respectively^32^. A low threshold age provides a narrow age-interval for reduction in premature mortality. So, a lag of ten and more years in the threshold age restraints the affirmative contribution of premature ages and moreover, augments negative repercussions of middle and old ages on mortality reductions in developing countries. As a consequence, a reduction in the premature mortality is subdued and the structural changes in causes of death is constrained. Over and above that, developing country India has the challenge of economic cost for the causes of death surveillance^33^, along with the cognisance of health transition^34^ and morbidity expansion^35^.

Smits and Monden^36^ demonstrates that the diffusion hypothesis is more conclusive while it is contingent upon the reduction in premature mortality. The oversimplification of the Omran’s model is not justified in the cognisance of the welfare Kuznets curve^37^. A developing country India do achieve on the higher survival of infants, children, and mothers^38^ to reducing premature mortality through long-term health programmes and policies^39^. However, NCDs intruded in the mid-1990s, and since thereupon premature mortality becomes a big concern in adult through old ages leading to dual burden of diseases in India^40^. Inevitably, the country attests a languid progression in mortality compression^27^ enduring large premature mortality and slow mortality deceleration^41,42^. Therefore, it is crucial to examine the role of the heavy burden of chronic NCDs^43,44^ and also injuries^45^ over a wide age range in adult through old ages for the progression in epidemiological transition in India.

The study assesses the role of inequality in age at death by causes of death for advances in epidemiological transition in India during a period of 30 years between 1990–1994 and 2015–2019. We tested the hypotheses that (a) whether chronic NCDs versus communicable diseases (CDs) contributes to higher inequality in age at death, (b) whether mortality decline in adult and higher age groups is crucial for structural changes in causes of death in high versus low mortality regimes; and, together they construe the prolonged dual burden of disease in India. The specific objectives of the study are (1) to assess the age- and cause-specific contribution to the changes in life expectancy at birth (Δe_0_) and inequality in age at death (ΔG_0_) and (2) to examine the transformation in age at death by age, sex, and causes of death. The study aims to explore the phenomenal phase of dual burden of disease by inequalities in mortality by causes of death and hence comprehends the progress of epidemiological transition in India.

## Data and methods

### Data

The age-cause-specific death rates (ACSDR) for 21 causes of death (level 2) by quinquennial age groups up to 95 + years and sex were retrieved from Global Burden of Disease (GBD)^46^ for the entire period of 1990–2019 (Supplementary Figs. S1 and S2). The causes of death are mapped with International Classification of Diseases (ICD) 10 classification^47^ in GBD data. There has been concern and issues for the availability of data and quality of data on causes of death for many countries as obstacles for computing mortality estimates^48–51^. The GBD data provides mortality estimates adjusted for systematic biases or inaccurate reporting for many countries and is also comparable across regions and time.

### Methods

#### Construction of abridged life tables

Abridged life tables were constructed using the Chiang method^52,53^, based on five-year moving average of ACSDR of 21 causes of death and overall mortality rate, by sex, in the studied period. Chiang method is based on the derivation of relation for the total number of person-years lived between exact ages *x* and *x* + *n* (${{}_{n}\mathrm{L}}_{\mathrm{x}})$ in terms of the average number of years lived by an individual of age *x* who dies in the interval (*x, x* + *n*) $({{}_{n}\mathrm{a}}_{\mathrm{x}}$). The columns of the life table are obtained using the following formulas:

${{}_{n}\mathrm{q}}_{\mathrm{x}}:$ probability of dying between age *x* and *x* + *n*

$${}_{n}{\text{q}}_{{\text{x}}} = \frac{{n*\left( {{}_{n}{\text{M}}_{{\text{x}}} } \right)}}{{1 + \left( {n - {}_{n}{\text{a}}_{{\text{x}}} } \right) * {}_{n}{\text{M}}_{{\text{x}}} }}$$

${\mathrm{l}}_{\mathrm{x}}:$ number of people alive at the exact age *x* among a hypothetical birth cohort of 100,000, usually called the radix of the life table.

$${\mathrm{l}}_{\mathrm{x}+\mathrm{n}}={\mathrm{l}}_{\mathrm{x}}*\left(1-{{}_{n}\mathrm{q}}_{\mathrm{x}}\right)$$

${{}_{n}\mathrm{d}}_{\mathrm{x}}:$ number of deaths in the age interval *x* to *x* + *n*

$${{}_{n}\mathrm{d}}_{\mathrm{x}}={\mathrm{l}}_{\mathrm{x}}*{{}_{n}\mathrm{q}}_{\mathrm{x}}$$

${{}_{n}\mathrm{L}}_{\mathrm{x}}:$ total number of person-years lived between exact ages *x* and *x* + *n*

$${{}_{n}\mathrm{L}}_{\mathrm{x}}=n*({\mathrm{l}}_{\mathrm{x}}- {{}_{n}\mathrm{d}}_{\mathrm{x}}+{{}_{n}\mathrm{a}}_{\mathrm{x}}*{{}_{n}\mathrm{d}}_{\mathrm{x}} )$$

${{}_{n}\mathrm{a}}_{\mathrm{x}}:$ average number of years lived in the age interval *x* to *x* + *n*

$${}_{n}{a}_{x}=\frac{{}_{n}{\mathrm{L}}_{x}-\mathrm{n}*{l}_{x+n}}{{{}_{}l}_{x}-{l_{x+n}}}$$

${\mathrm{T}}_{\mathrm{x}}:$ total number of person-years lived beyond Age *x*

$${\mathrm{T}}_{\mathrm{x}}={\mathrm{T}}_{\mathrm{x}+\mathrm{n}}+{{}_{n}\mathrm{L}}_{\mathrm{x}}$$

${\mathrm{e}}_{\mathrm{x}}$: average number of years of life remaining for a person alive at the beginning of age interval x.

$${\mathrm{e}}_{\mathrm{x}}=\frac{{\mathrm{T}}_{\mathrm{x}}}{{\mathrm{l}}_{\mathrm{x}}}.$$

#### Inequality in age at death

The Gini coefficient at birth (G_0_) and the disparity in lifespan (e^†^) are often applied in the field of demography. G_0_ measures the variability in age at death. Whereas, e^†^ measures life years lost due to deaths. Wilmoth and Horiuchi^54^ and Vaupel, et al.^32^ have shown that many inequality measures, including the Gini, are highly correlated. We applied G_0_^22^ as a measure of inequality in age at death because it satisfies the four fundamental properties, i.e. the Pigou-Dalton transfer principle, scale invariance, population variance, and symmetry for an inequality measure^23^, and so, is a preferred measure of inequality. The G_0_ for an abridged life table is expressed as:

$${G}_{0}=1-\frac{1}{{{e}_{0}{*[l}_{0}]}^{2}}{\sum }_{x=0}^{w-1}n*\left[{{(l}_{x+n})}^{2}+{{\hat{{\text{a}}} }}_{x}\left({{(l}_{x})}^{2}-{{(l}_{x+n})}^{2}\right)\right]$$

where,

$${{\hat{{\text{a}}} }}_{x}=\frac{1-\frac{2}{3}{q}_{x}{{+}C}_{x}\left(2{-q}_{x}-{\frac{6}{5}C}_{x}\right)}{2-{q}_{x}}$$

$${c}_{x}={\mathrm{a}}_{x}-\frac{1}{2}$$

$${{}_{1}\hat{{\text{a}}} }_{0}={}_{1}{\mathrm{a}}_{0}\left(1{{-}_{1}q}_{0}\frac{3+0.831{*}_{1}{a}_{0}}{2{{+}_{1}q}_{0}}\right)$$

${\widehat{a}}_{x}$ is the adjusted ${a}_{x}$ for deviation in the pace of ${{}_{n}q}_{x}$ by age, and ${a}_{x}\left[=\frac{\left({{}_{n}L}_{x}/n\right)-{l}_{x+n}}{({l}_{x}-{l}_{x+n})}\right]$ is the person-years lived by the individuals who have died within the given interval.

#### Decomposition of e_0_ and G_0_ using the replacement method

Yadav, et al.^55^ shows that discrete^56^ and replacement^57^ methods of decomposition analyses^23,58^ produce similar results for e_0_ and G_0_. The difference of quantities between two populations is shown as

$$\Delta {e}_{0},\Delta {G}_{0}=\sum_{i=0}^{n-1}\left({\in }_{0,{x}_{i+1}}-{\in }_{0,{x}_{i}}\right)=\sum_{i=0}^{n}{\in }_{i}$$

and,

$${\in }_{i}={e}_{0}[{M}^{\left({x}_{i}\right)}]-{e}_{0}^{\mathrm{^{\prime}}}{[M}^{\left({x}_{i}\right)}],{G}_{0}[{M}^{\left({x}_{i}\right)}]-{G}_{0}^{\mathrm{^{\prime}}}{[M}^{\left({x}_{i}\right)}].$$

where, ${M}^{({x}_{i})}$ is a vector of age-specific death rates (ASDR) with elements $m^{\prime}_{x}$ for *x* <  = *x*_*i*_ and ${m}_{x}$ for *x* >  = *x*_*i*_.

#### Decomposition of e_0_ and G_0_ by causes of death and age groups

Specifically, the contribution of $${j}{th}$$ cause of death to the contribution $${\in }_{i}$$ in $${i}{th}$$ age-interval [$$x, x+n)$$ is calculated as

$${\in }_{i}^{j}=\left(\frac{{{}^{a}m}_{{x}_{i}|{x}_{i+n}}^{j}-{{}^{b}m}_{{x}_{i}|{x}_{i+n}}^{j}}{{{}^{a}m}_{{x}_{i}|{x}_{i+n}}-{{}^{b}m}_{{x}_{i}|{x}_{i+n}}}\right){\in }_{i}$$

where, $${{}^{a}m}_{{x}_{i}|{x}_{i+n}}^{j}$$ and $${{}^{b}m}_{{x}_{i}|{x}_{i+n}}^{j}$$ are ACSDR of $${j}{th}$$ cause of death in $${i}{th}$$ age-interval [$$x, x+n)$$ in the population $$a$$ and $$b$$, respectively, and $${{}^{a}m}_{{x}_{i}|{x}_{i+n}}$$ and $${{}^{b}m}_{{x}_{i}|{x}_{i+n}}$$ are ASDR in $${i}^{th}$$ age-interval [$$x, x+n)$$ in the population $$a$$ and $$b$$, respectively. The decomposition analysis provides age-cause-specific contributions to Δe_0_ and ΔG_0_ for both sexes, which are comparable across population subgroups and over time. The age group of 85+ years is the last age group presented for any level of presentation. Also, we have considered neonatal disorders and maternal disorders as separate diseases, so 22 causes of death in total.

### Ethical approval

We confirm that all methods were carried out in accordance with relevant guidelines and regulations.

## Results

### Age-specific contributions to Δe_0_ and ΔG_0_, India, 1990–2019

**Figure 1:**
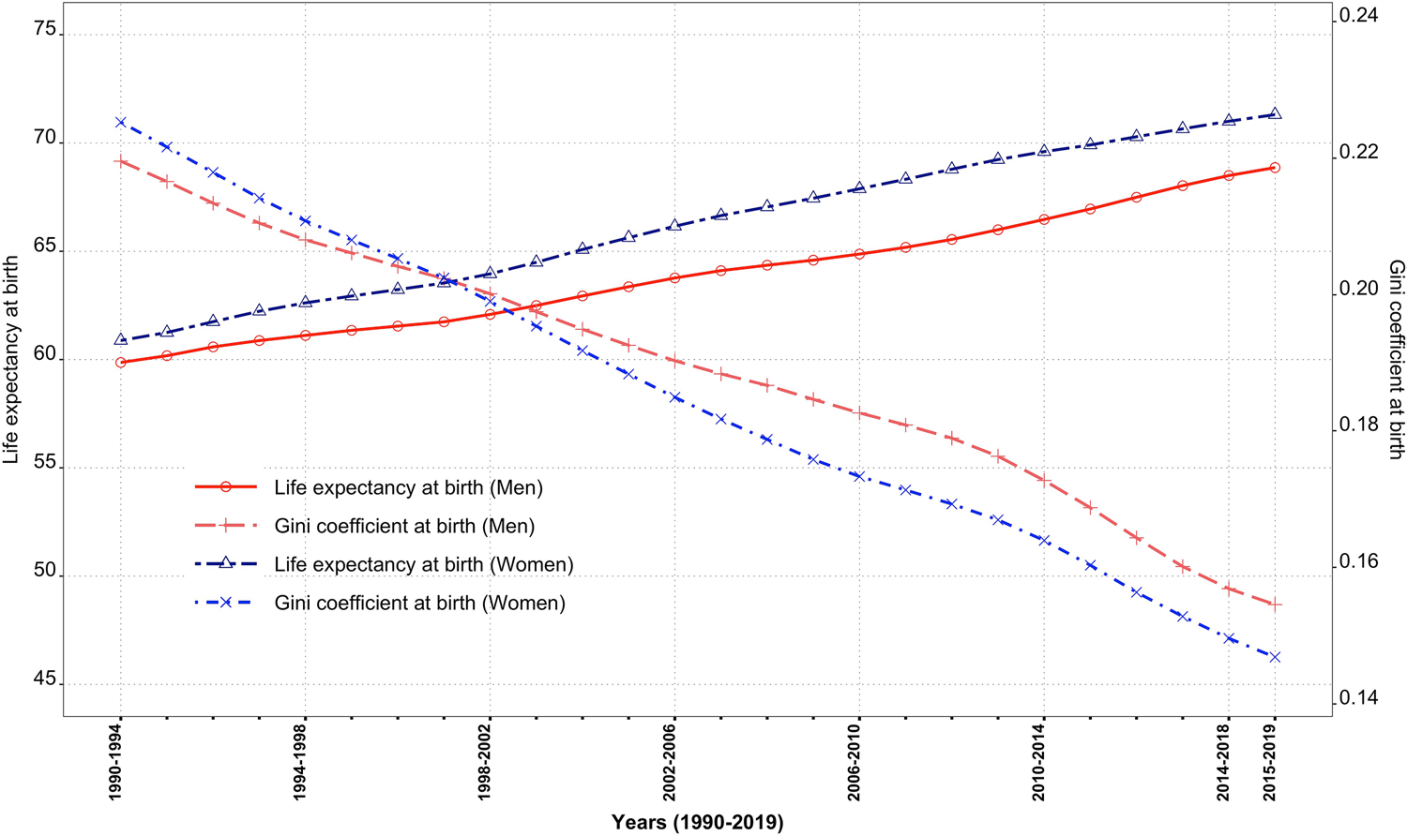
Trends in e_0_ and G_0_, men and women, 1990–2019.

**Figure 2:**
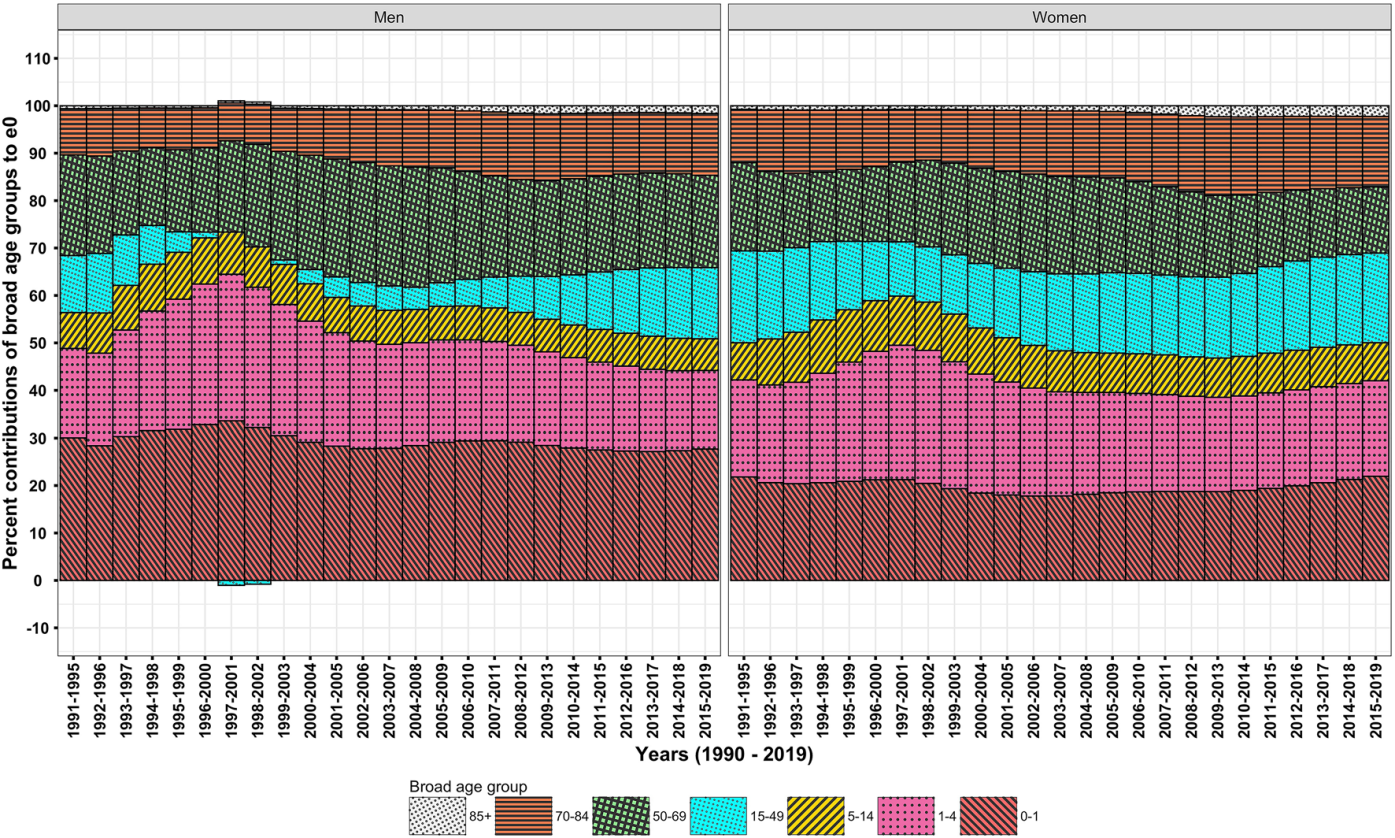
Temporal changes in age-specific contributions to Δe_0_, men and women, 1990–2019.

**Figure 3:**
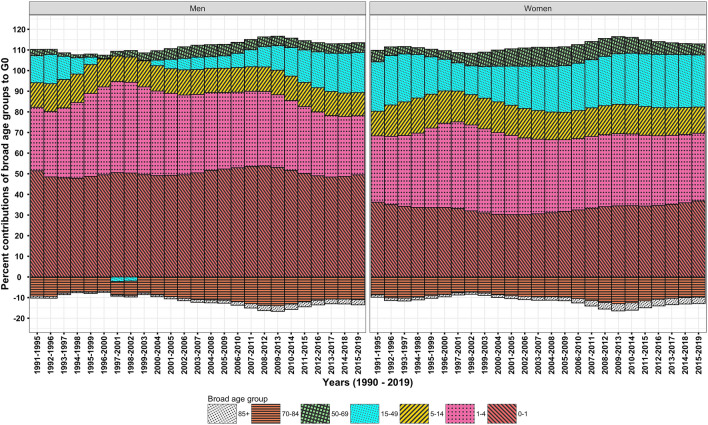
Temporal changes in age-specific contributions to ΔG_0_, men and women, 1990–2019.

The e_0_ for men and women increased from 59.8 and 60.8 years in 1990–1994 to 68.8 and 71.3 years, respectively, during 1990–2019. Contemporaneously, the G_0_ values for men and women declined respectively from 0.219 and 0.225 in 1990–1994, 0.188 and 0.181 in 2003–2007, and to 0.154 and 0.146 in 2015–2019 (Fig. 1). Men compared to women show higher G_0_ values since the mid-2000s. A higher G_0_ value confirms high uncertainty in age at death in men than in women. The decomposition analysis of these G_0_ values by quinquennial age groups reveals a huge equalising contribution of 78.1 and 69.6% to ΔG_0_ (Fig. 3, Table 1) by male and female in 0–4 years, respectively, which is approximately one-and-a-half times than that to Δe_0_ (Fig. 2, Table 1). It testifies that decline in G_0_ values is majorly driven by mortality reductions among infants and children.

**Table 1:** Age-specific per cent contributions to Δe_0_ and ΔG_0_, men and women, India, 1990–1994 and 2015–2019.

Age groups	Δe_0_		ΔG_0_	
	Men (9 years)	Women (10.5 years)	Men (− 0.065)	Women (− 0.079)
0–1	27.7	21.9	49.5	36.8
1–4	16.5	20.1	28.6	32.8
5–9	4.8	5.8	8.1	9.3
10–14	1.9	2.2	3.1	3.4
15–19	2.6	3.4	4.1	5.2
20–24	2.3	4.0	3.5	5.8
25–29	1.8	2.9	2.6	4.1
30–34	1.7	2.6	2.2	3.4
35–39	1.3	2.2	1.6	2.7
40–44	2.1	1.9	2.4	2.1
45–49	3.3	1.9	3.1	1.9
50–54	3.8	1.5	2.8	1.3
55–59	3.5	3.2	1.7	2.1
60–64	6.0	4.0	1.2	1.6
65–69	6.2	5.3	− 1.0	0.4
70–74	5.8	5.8	− 3.2	− 1.8
75–79	4.7	5.8	− 4.5	− 4.2
80–84	2.5	3.3	− 3.3	− 3.8
85 +	1.6	2.3	− 2.5	− 3.2

Source: Own calculations.

However, men and women in their adult age group of 15–49 years have shown most dramatic temporal changes from almost *zero* or negative contribution in the late 2000s and then manoeuvre it to considerable values of 19.4 and 25.4% to ΔG_0_ (Fig. 3) and 15 and 18.8%, respectively, to Δe_0_ in the late 2010s (Fig. 2). This skimpy, meagre contribution of adult ages in men has played a major role for the sex differentials in e_0_ and G_0_ negatively skewed towards men.

The discordance between age-specific contributions to Δe_0_ and ΔG_0_ are apparent in the middle (50–69 years) and old (70 + years) age groups. The contribution of 19.5 and 14%, respectively, to Δe_0_ by men and women in middle age group is approximately three-folds than that to ΔG_0_. It shows a quantum leap for Δe_0_ but not for ΔG_0_. On the other hand, a disequalising (negative) contribution of − 14.5 and − 12.9% to ΔG_0_ by men and women in old ages, respectively, signify a disparate role not manifested in the decomposition analysis of Δe_0_ (Table 1). These large disequalising contributions impede the progression in G_0_, hence the phenomenon of high e_0_ and low G_0_ is restrained.

The scrutinization of outputs reveals greater, significant role of infant and child age groups for changes in G_0_ and e_0_ for the entire period of 1990–2019; however, to take note, their age-specific contributions to ΔG_0_ and Δe_0_ has more or less remained unchanged over time. Rather, temporal changes in age-specific contributions at adult (15–49 years) especially in men are conspicuous. Further, the middle and old age groups contributed variably to Δe_0_ and ΔG_0_, but, their temporal contribution has moderately changed. In sum, India’s *pattern* of age-specific contributions to Δe_0_ or ΔG_0_ has remained more or less similar during studied period; nonetheless, the *pattern* has shown encouraging mortality changes at adult ages since the mid-2000s.

### Dominance of chronic NCDs versus CDs, India, 1990–2019

**Figure 4:**
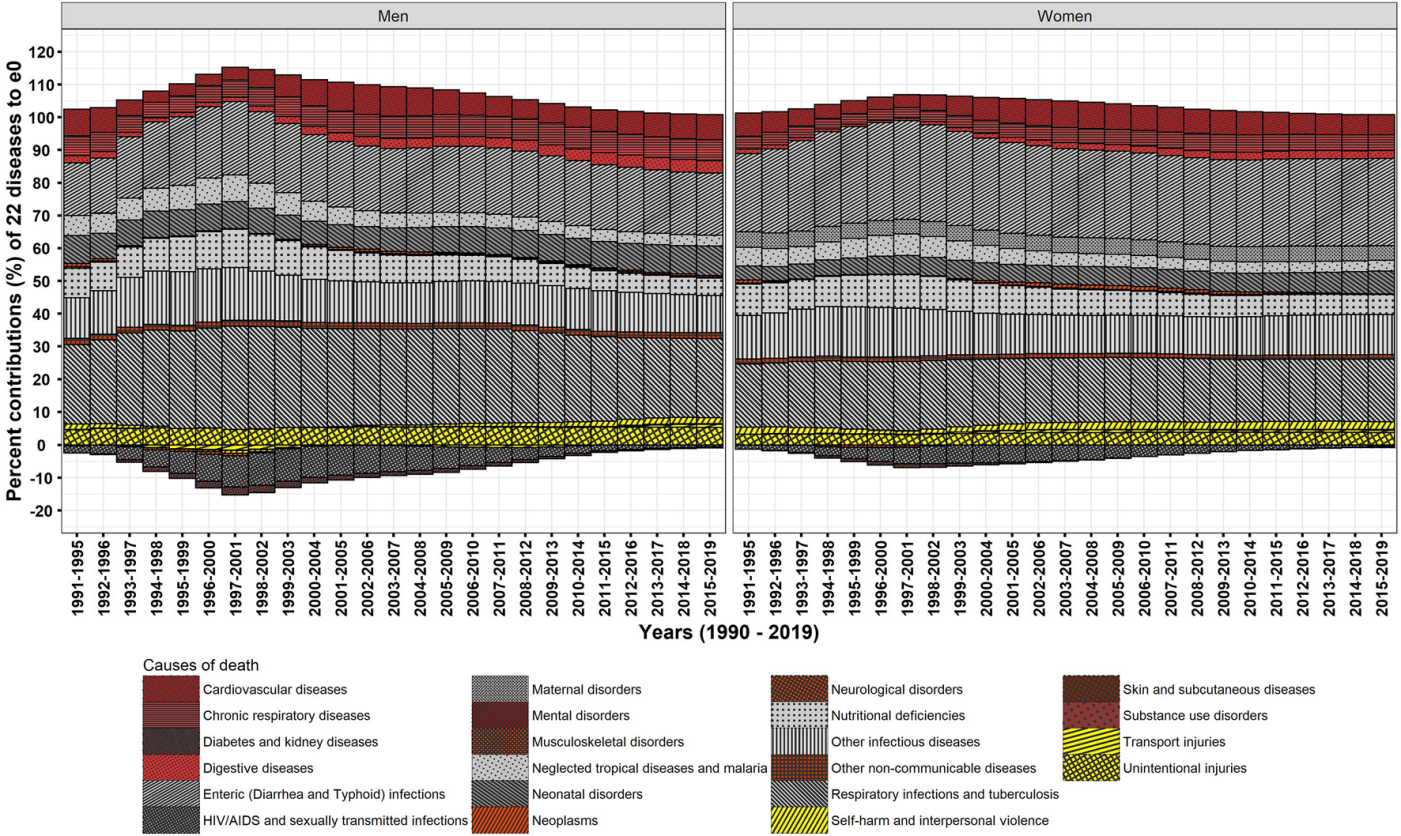
Temporal changes in cause-specific contributions to Δe_0_, men and women, 1990–2019.

**Figure 5:**
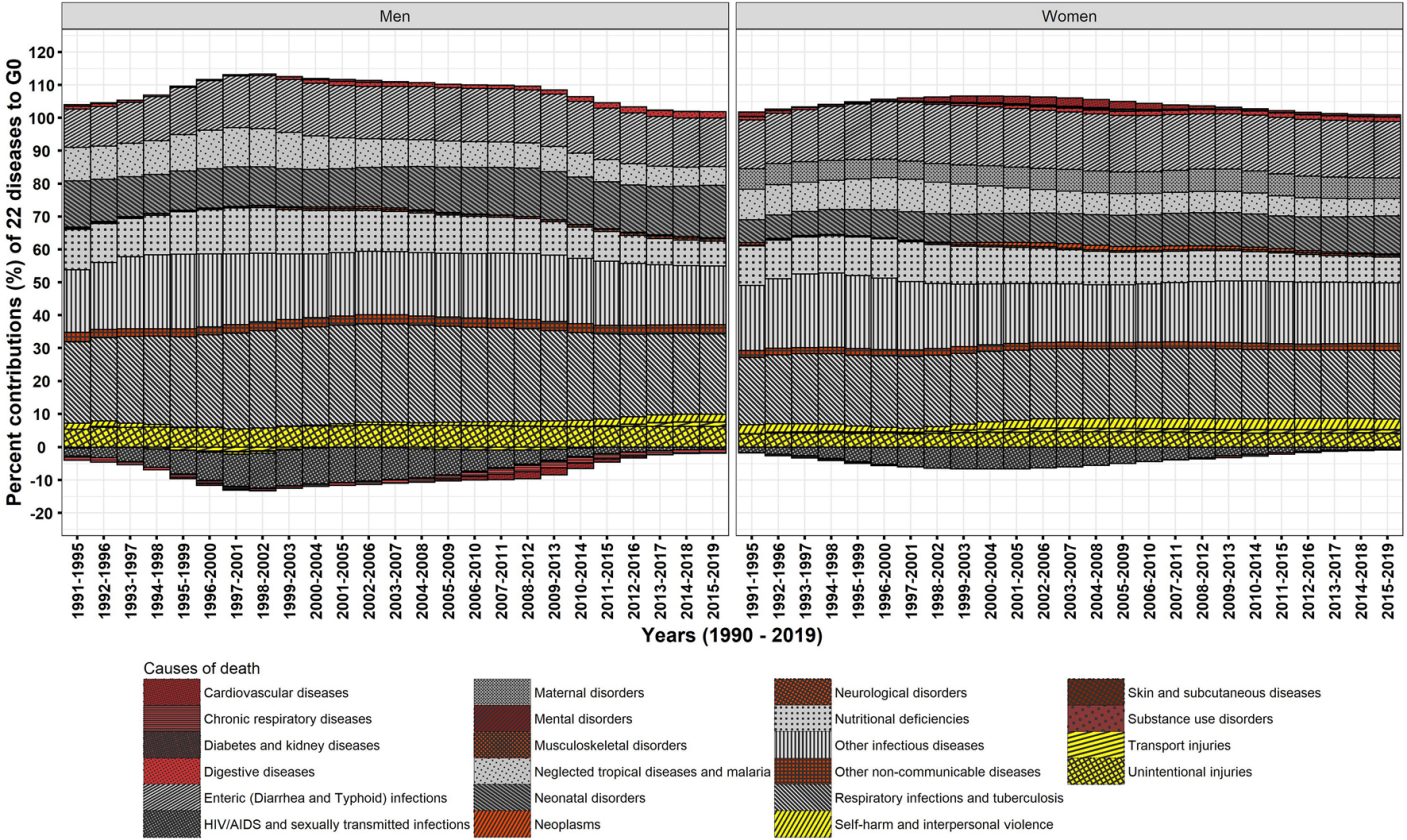
Temporal changes in cause-specific contributions to ΔG_0_, men and women, 1990–2019.

The decomposition analysis by causes of death demonstrate that communicable diseases (CDs), NCDs, and injuries contributed respectively 85.6, 4.6, and 9.8% in men and 86.8, 4.8, and 8.3% in women to ΔG_0_ between 1990–1994 and 2015–2019 (Table 2). The contributions to ΔG_0_ by NCDs were larger when compared to that of Δe_0_. The results show that CDs committedly reshapes the distribution of age at death, whereas NCDs hardly matters for equalising age at death. Exploring by 22 causes of death (Figs. 4 and 5) reveals that the huge contribution by CDs in men and women comprises of the largest contribution of 24.4 and 20.9%, respectively, to ΔG_0_ by respiratory infections and tuberculosis. Respiratory infections and tuberculosis together with other infectious diseases, enteric infections, and neonatal and maternal disorders have contributed approximately 72.7 and 74.1% to ΔG_0_ in men and women, respectively. Temporal analysis reveals that the share of these four diseases have remained nearly stagnant to ΔG_0_ (Fig. 5) and Δe_0_ (Fig. 4) in the studied period. This nearly stagnant contributions confirm a consistent mortality decline for these four diseases. In addition to that, the share of neonatal disorders in female children has been approximately doubled from 6.9 in 1990–1994 to 11.4% to ΔG_0_ in 2015–2019, confirming a mortality decline among female children. However, the share of neglected tropical diseases and malaria and nutritional deficiencies reduced by half to ΔG_0_ during 1990–2019 because of mortality increase. Of great importance, these CDs manoeuvre a low dispersion in the distribution of age at death and guides the causes of death structure.

**Table 2:** Per cent contributions of causes of death to Δe_0_ and ΔG_0_, men and women, India, 1990–1994 and 2015–2019.

Broad category	Causes of death	Δe_0_		ΔG_0_	
		Men (9 years)	Women (10.5 years)	Men (− 0.065)	Women (− 0.079)
CDs	Enteric (Diarrhea and Typhoid) infections	19.0	26.6	14.7	17.1
CDs	HIV/AIDS and sexually transmitted infections	− 0.6	− 0.5	− 0.5	− 0.5
CDs	Neonatal disorders	9.0	6.9	15.8	11.4
CDs	Maternal disorders	–	4.5	–	6.2
CDs	Neglected tropical diseases and malaria	3.2	3.2	5.8	5.4
CDs	Nutritional deficiencies	5.3	6.1	7.5	7.9
CDs	Other infectious diseases	11.4	12.2	17.8	18.4
CDs	Respiratory infections and tuberculosis	24.1	19.1	24.4	20.9
**Communicable diseases (CDs)**		71.5	78.2	85.6	86.9
Inj	Self-harm and interpersonal violence	1.9	2.4	2.3	3.2
Inj	Transport injuries	0.9	0.7	1.3	0.9
Inj	Unintentional injuries	5.3	3.8	6.2	4.2
**Injuries (Inj)**		8.1	6.9	9.8	8.3
NCDs	Cardiovascular diseases	7.7	6.2	0.0	0.6
NCDs	Chronic respiratory diseases	6.4	4.8	− 1.3	− 0.4
NCDs	Diabetes and kidney diseases	− 0.2	− 0.2	0.0	0.1
NCDs	Digestive diseases	3.8	2.4	1.9	1.4
NCDs	Mental disorders	0.0	0.0	0.0	0.0
NCDs	Musculoskeletal disorders	0.0	0.0	0.0	0.0
NCDs	Neoplasms	0.5	− 0.1	0.4	0.4
NCDs	Neurological disorders	0.4	0.3	0.7	0.6
NCDs	Other non-communicable diseases	1.8	1.4	2.8	2.1
NCDs	Skin and subcutaneous diseases	0.1	0.1	0.1	0.1
NCDs	Substance use disorders	0.1	0.0	0.0	0.0
**Noncommunicable diseases (NCDs)**		20.6	14.9	4.6	4.9

Source: Own calculations.

HIV/AIDS and sexually transmitted infections is the only disease among CDs, which had shown negative contribution to Δe_0_ as well as disequalising contribution to ΔG_0_ for more than 20 years between the mid-1990s and the mid-2010s. Nonetheless, this negative contribution of HIV/AIDS and sexually transmitted infections reduced to ~ − 0.5% in 2015–2019, with the rapid decline in its prevalence and mortality rate.

Among injuries, unintentional injuries in men and women have shown small contributions of 6.2 and 4.2% to ΔG_0_, respectively. Other two causes of death, namely self-harm and interpersonal violence and transport injuries, showed very small contributions (Table 2).

Compared to CDs and injuries, NCDs have shown small contribution to Δe_0_ and negligible contribution to ΔG_0_. Chronic respiratory diseases, mainly comprise of chronic obstructive pulmonary disease (COPD), asthma, and pneumoconiosis, and cardiovascular diseases, mainly comprise of hypertensive heart disease and strokes, have shown contributions in the range of 6–8% to Δe_0_; however, they showed negligible contributions to ΔG_0_. Specifically, chronic respiratory diseases and cardiovascular diseases in men has shown disequalising contributions to ΔG_0_ between the mid-2000s to the mid-2010s which indicates expansion of deaths over age and time.

In sum, NCDs shows insignificant effect on the dispersion in age at death besides small contribution to Δe_0_ that corroborates a shift in age at death. Negligible contributions by many causes of death among NCDs to ΔG_0_ intrinsically negate their small contributions to Δe_0_. Thus, NCDs has shown an insufficient contribution and attests its aftermath by impeding the progression in G_0_. Further, the dominance of NCDs at adult and higher ages is also upheld by the modest adult-, middle-, and old-age mortality decline.

### Transformation in distribution of age at death by causes of death, India, 1990–2019

**Figure 6:**
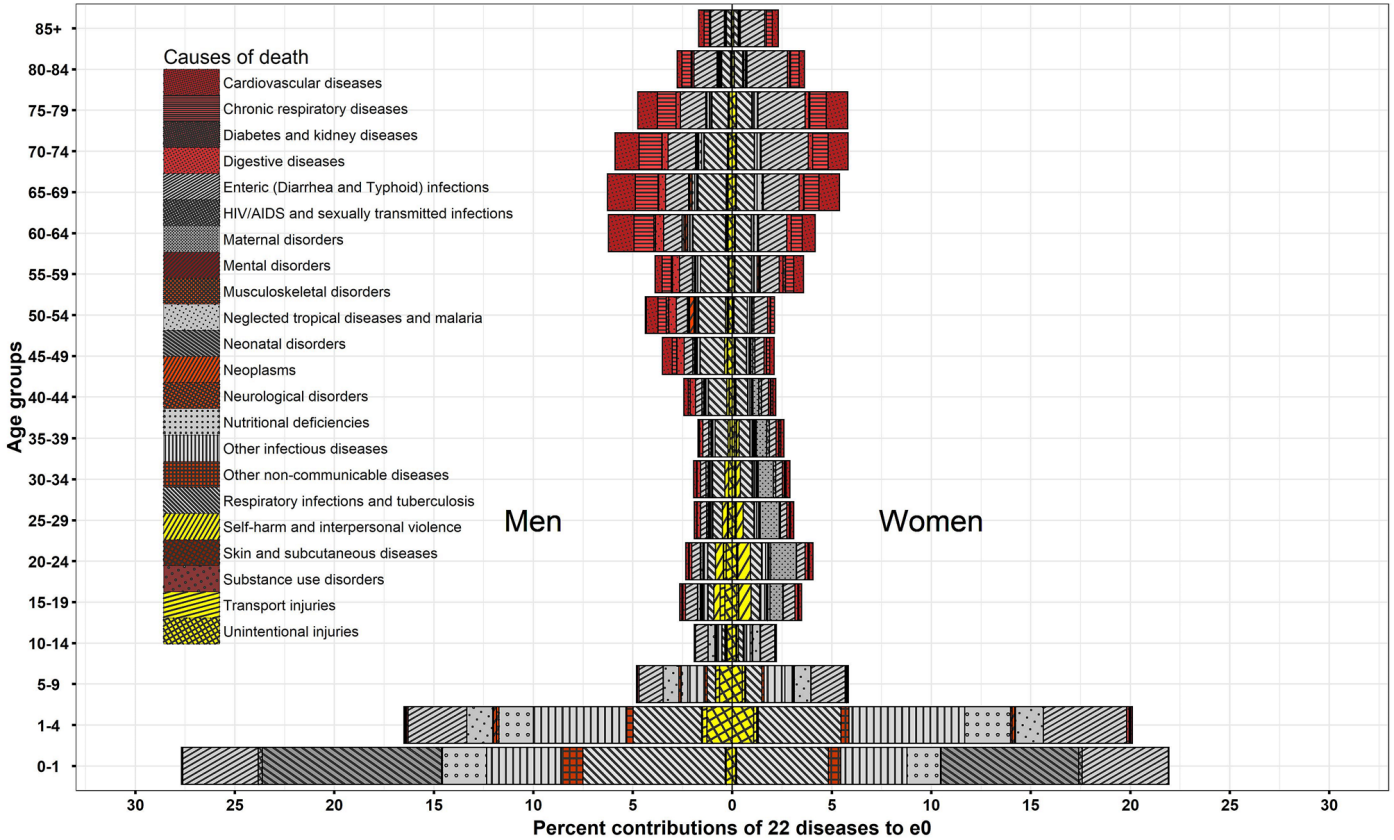
Age-cause-specific contributions to Δe_0_, men and women, between 1990–1994 and 2015–2019.

**Figure 7:**
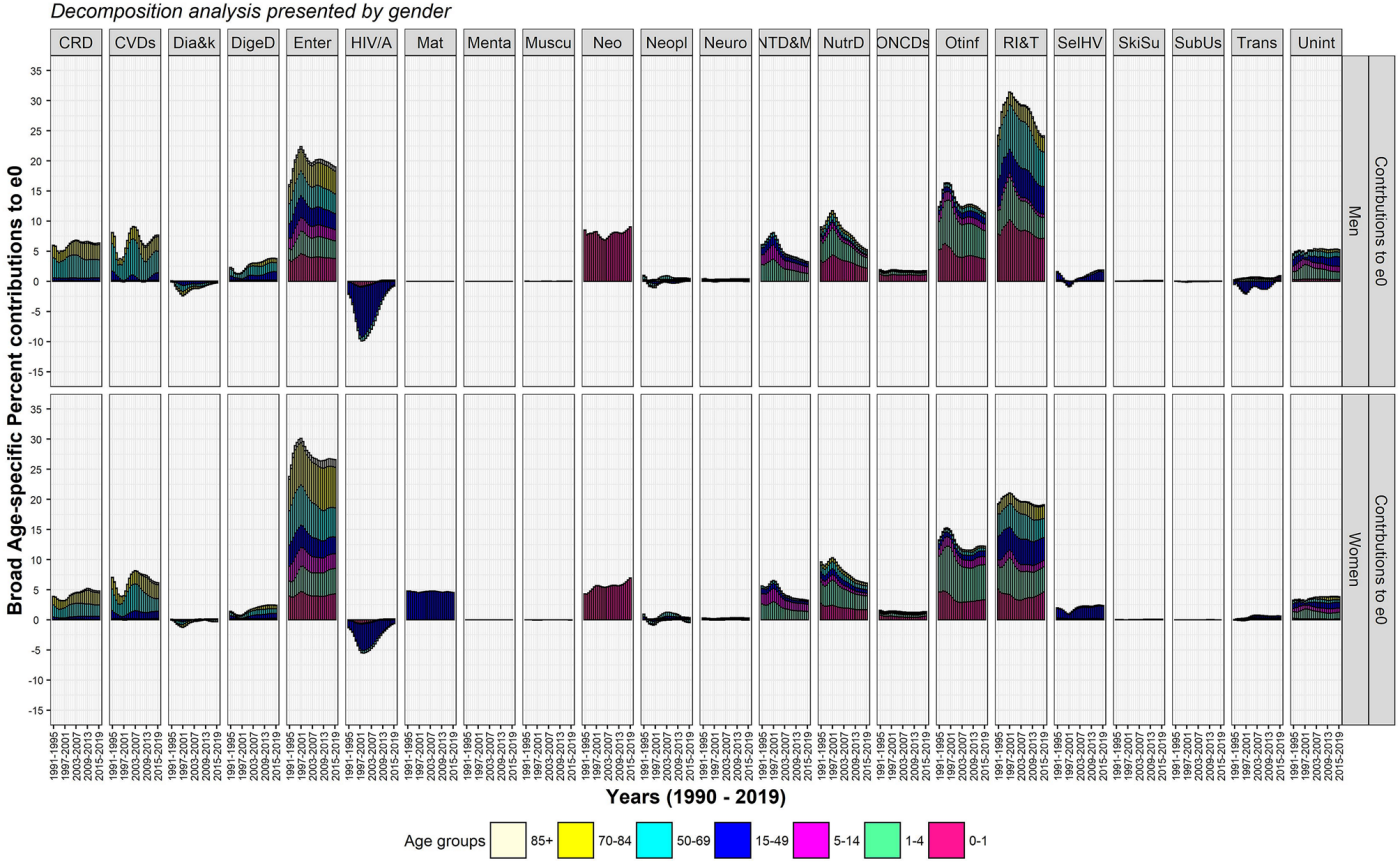
Temporal changes in age-cause-specific contributions to Δe_0_, men and women, India, 1990–2019. *CRD*, chronic respiratory diseases, *CVDs* cardiovascular diseases, *Dia&k* diabetes and kidney diseases, *DigeD* digestive diseases, *Enter* enteric (diarrhea and typhoid) infections, *HIV/A* HIV/AIDS and STI, *Mat* maternal disorders,* Menta* mental disorders, *Muscu* musculoskeletal disorders, *Neo* neonatal disorders, *Neopl* neoplasms, *Neuro* neurological disorders, *NTD&M* neg. tropical diseases and malaria, *NutrD* nutritional deficiencies, *ONCDs* other noncommunicable diseases, *Otinf* other infectious diseases, *RI&T* respiratory infections and tuberculosis, *SelHV* self-harm and interpersonal violence, *SkiSu* skin and subcutaneous diseases, *SubUs* substance use disorders, *Trans* transport injuries, *Unint* unintentional injuries.

**Figure 8:**
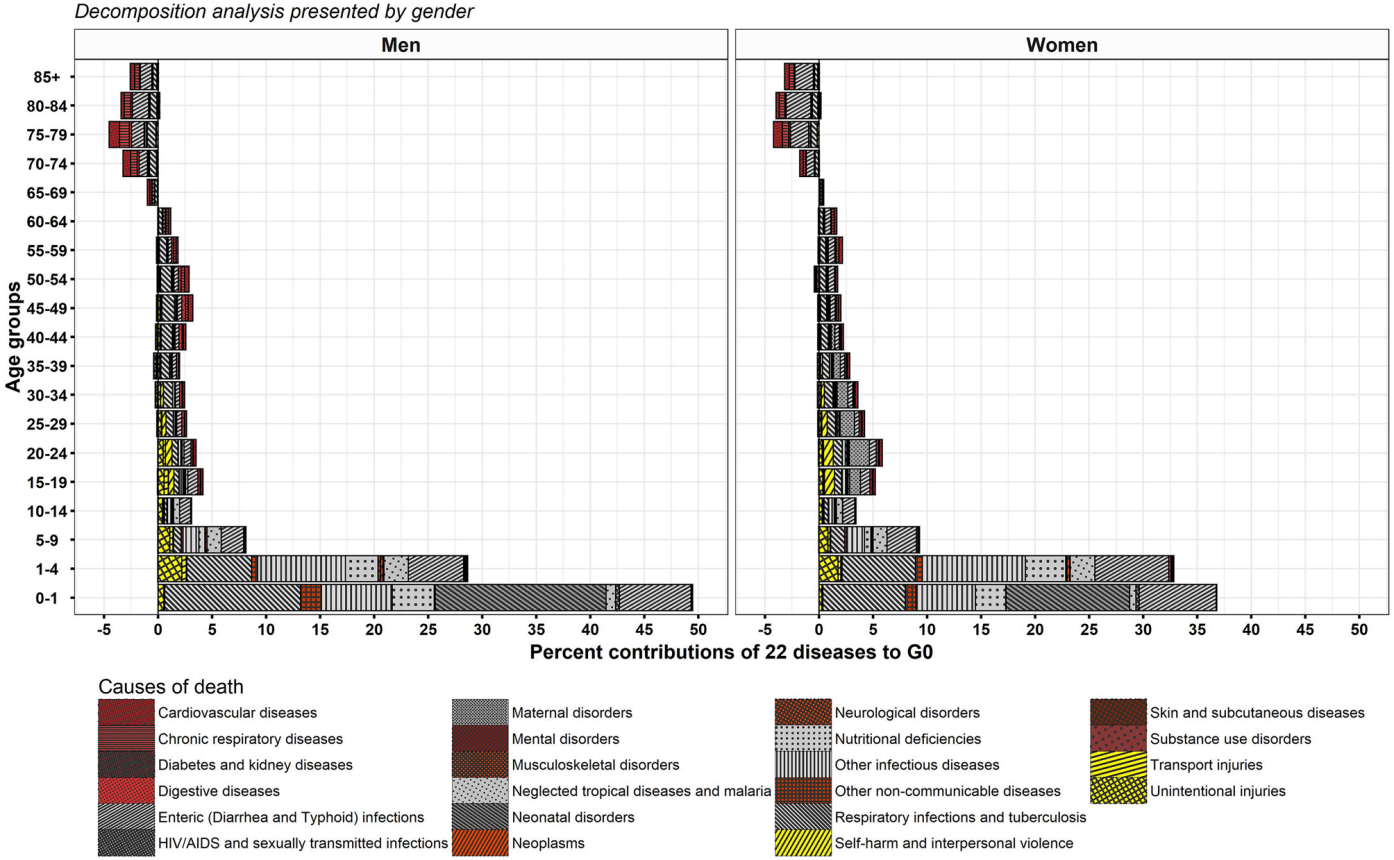
Age-cause-specific contributions to ΔG_0_, men and women, between 1990–1994 and 2015–2019.

**Figure 9:**
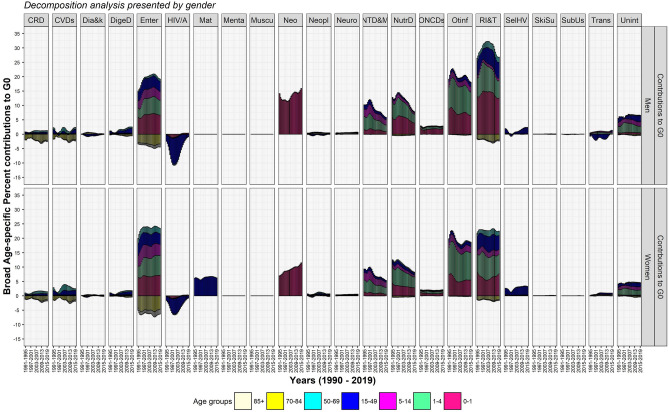
Temporal changes in age-cause-specific contributions to ΔG_0_, men and women, India, 1990–2019. *CRD*, chronic respiratory diseases, *CVDs* cardiovascular diseases, *Dia&k* diabetes and kidney diseases, *DigeD* digestive diseases, *Enter* enteric (diarrhea and typhoid) infections, *HIV/A* HIV/AIDS and STI, *Mat* maternal disorders,* Menta* mental disorders, *Muscu* musculoskeletal disorders, *Neo* neonatal disorders, *Neopl* neoplasms, *Neuro* neurological disorders, *NTD&M* neg. tropical diseases and malaria, *NutrD* nutritional deficiencies, *ONCDs* other noncommunicable diseases, *Otinf* other infectious diseases, *RI&T* respiratory infections and tuberculosis, *SelHV* self-harm and interpersonal violence, *SkiSu* skin and subcutaneous diseases, *SubUs* substance use disorders, *Trans* transport injuries, *Unint* unintentional injuries.

India during the period of 1990–2019 shows an impressive pattern of age-specific contributions mainly dominated by CDs in infant through old ages (Figs. 6, 7, 8, and 9). Infants show reduction in their toll of deaths caused by neonatal disorders which have been most crucial for a shorter toe of the tilted *j*-shaped age pattern of mortality (see Supplementary Figs. S1 and S2) and thus, to a more left-skewed unimodal distribution of age at death. This transformation was also sustained by the reduction in the burden caused by respiratory infections and tuberculosis, enteric infections, other infectious diseases, and nutritional deficiencies in infant as well as child and adolescent age groups (Figs. 7 and 9).

In the later stages of life, i.e. from adult to old ages, respiratory infections and tuberculosis and enteric infections show their distinguishable contributions to Δe_0_ and ΔG_0_ (Figs. 6 and 8). Adult men and adult women show respectively 4.5 and 9.2% whereas middle-aged men and middle-aged women show 2.3 and 3.7% reduction in toll of deaths caused by respiratory infections and tuberculosis and enteric infections for a better G_0_ (Figs. 8 and 9). Adult women did experience an aversion of 6.5% maternal deaths in addition to whatsoever reckoned in adult men (Figs. 7 and 9). Contrary to these equalising effects by many CDs, HIV/AIDS and sexually transmitted diseases exhibited disequalising effect on ΔG_0_ by adult men and adult women which lessens the equalising contribution in adult age group for a span of time (Fig. 9).

Men and women in their middle-age group have shown small contributions of 2.3 and 3.7% to ΔG_0_, respectively, by respiratory infections and tuberculosis together with enteric infections. Compared to these two predominant diseases, cardiovascular diseases plus chronic respiratory diseases for men and women respectively have shown smaller contribution of 0.8 and 1.7% in adult age group and 1.1 and 1.8% in the middle age group to ΔG_0_ (Figs. 7 and 9). In sum, these four causes of death contributed on an average 3.3 and 5.5% to ΔG_0_ (Fig. 9) whereas 17.8 and 14.6%, respectively, to Δe_0_ (Fig. 7) by men and women in their middle ages. These large contribution to Δe_0_ by enteric infections and respiratory infections and tuberculosis at middle ages attested deceleration in mortality rates (Supplementary Fig. S1), but a small contribution to ΔG_0_ at the same ages reveals negligible changes in the distribution of age at death. The middle-aged persons survived and delayed their deaths; thus, they have been instrumental for a shift in the distribution of age at death by adding a few points to e_0_ but insignificantly contribute to its dispersion. Thus, its insufficient contribution for a change in dispersion curbs the progression in G_0_.

The progression in G_0_ is further opposed by the three-fold disequalising effect for both men and women in their old age group of 70 + years in comparison to that of middle age group by the same causes of death, i.e. respiratory infections and tuberculosis, enteric infections, cardiovascular diseases and chronic respiratory diseases. By causes of death, the disequalising effect in 70 + years is mainly contributed by enteric infections; on an average, a contribution of − 4.2 and − 6.4% to ΔG_0_ in men and women, respectively, during 1990–2019 (Fig. 9). The disequalising effect in old men and old women were approximately − 1.5 and − 2% to ΔG_0_, respectively, for each of cardiovascular diseases, chronic respiratory diseases, and respiratory infections and tuberculosis and enteric infections (Fig. 9). The disequalising contributions by these dominant causes of death at old ages are a consequence of a higher mortality rates with minor changes at old ages (Figs. 8 and 9). Also, disequalising contribution at old ages in India remains large because of senility and low threshold age.

To note, the chronic NCDs, enteric infections and respiratory infections and tuberculosis showed larger disequalising contributions in old age group than small equalising contributions in the middle age group. So, the net contribution by chronic NCDs and enteric infections and respiratory and infectious diseases in higher age groups is negative. Also, other causes of death showed negligible contributions in middle and old age groups. It implies that a wide age-interval comprising middle and old ages in India is not at all contributing to the progression in G_0_.

The outcomes reveal that the transformations in age at death are majorly contributed by enteric infections followed by respiratory diseases and infectious diseases. CDs have been contributory for a shift in e_0_ as well as reshaping the distribution of age at death despite its lower mortality rate than that of NCDs during the studied period. Contrary to the remarkable role of CDs, many NCDs did not contribute enough to equalising age at death in adult through old ages; however, they corroborate for a trivial shift in e_0_. Nonetheless, a greater change in CDs and a subtle change in NCDs testify the progression in the later phases of epidemiological transition.

Overall, the results reveal a worsening phase in epidemiological transition caused by chronic NCDs in adult, middle, and old age groups, with nearly unchanged toll of deaths caused by injuries in adult ages, with great contribution of mortality decline of CDs in infant through oldest of old ages. While the analyses of e_0_ conceals the reason for modest changes in cause of death structure, particularly for NCDs, the analyses of G_0_ apparently highlights negligible contribution in wide age-interval in middle through old ages. The outcomes unravel that progression in epidemiological transition is curbed by the high inequality in age at death caused by chronic NCDs in India. Higher inequalities in age at death contributed by the mortality pattern of chronic NCDs raises concern about the structural changes in causes of death not befitting the progression in epidemiological transition.

## Discussion

India transcended from the high mortality regime in the early 1990s to a low mortality regime in the late 2010s. Despite showing a significant decline in the burden of CDs mainly among infants and children during 1990–2019, the adults and olds are enduring the heavy burden of NCDs together with that of CDs. There are evidences of chronic NCDs contributing for a rise in e_0_ (Figs. 2 and 4); nonetheless, its role for causes of death structure explicitly examined by disparity in lifespan is more crucial. In particular, for India, mortality analysis by causes of death has largely remained unexplored. Acknowledging the gap, this study examines the inequality in mortality (Fig. 1) by 22 causes of death by performing the decomposition analyses^59^ to assess age-cause-specific contributions to the changes in life expectancy at birth (Δe_0_) and inequality in age at death (ΔG_0_), using GBD data for the entire period of 1990–2019. The study aims to examine the progress of epidemiological transition during a period of 30 years between 1990 and 2019.

The study outcomes reveal that many CDs rather than NCDs significantly contributed to Δe_0_ and ΔG_0_ (Table 2). A reduction in the burden of CDs in infant through old ages signify their greater role for a structural change in causes of death (Figs. 4 and 5). Amongst demographic age groups, infants have been the largest contributor to Δe_0_ and ΔG_0_ followed by children, adolescents, and women in their reproductive age groups (Figs. 2 and 3). The same is also corroborated by the rapid decline in infant mortality rate and under-five mortality (U5MR), and Maternal Mortality Ratio (MMR)^60^ over time. Further, the dramatic changes in the age-specific contributions by men in adult age group is bolstered by the reduction in the burden of HIV/AIDS and sexually transmitted diseases^61^ together with that of respiratory infections and tuberculosis and enteric infections. Enteric infections and respiratory infections and tuberculosis also show considerable contributions to Δe_0_ and ΔG_0_ at middle and old ages (Figs. 7 and 9).

However, many NCDs at middle through old ages contribute considerably to Δe_0_ but negligibly to ΔG_0_. The disparateness in the age-specific contributions to Δe_0_ (Figs. 6 and 7) and ΔG_0_ (Figs. 8 and 9) by CDs and NCDs at middle and old ages highlights the contrasts for a shift in e_0_ versus transformation in age at death as measured by G_0_. The mortality decline for CDs at infant through old ages showed a shift in e_0_ as well as reshaped the distribution of age at death; however, the mortality decline for NCDs at middle and old ages trivially corroborated the shift in e_0_ but importantly didn’t contribute to the transformation in age at death or its dispersion (G_0_) (Figs. 6, 7, 8, and 9). A negligible contribution to ΔG_0_ by NCDs at middle through old ages was on the account of its disequalising contributions.

By the virtue of equalising (positive) and disequalising (negative) effects on G_0_, the NCDs and CDs showing affirmative contributions before the threshold age, however, do not contribute for a better G_0_ after that threshold age^17,62^ (Figs. 8 and 9). The low threshold age in 65–69 years^62^ is critical for India (Fig. 9) because a possibility of reduction in premature deaths is restrained by a narrow age-interval. On the other hand, a wide age-interval at middle through old ages burdened with NCDs as well as CDs allows for high disparity in lifespan. Thus, a low threshold age puts major constraints for possible affirmative age-specific contributions from NCDs emplaced at middle and old ages. The same is also applicable for CDs, however, given their preponderance at infant through old ages they do have large reduction in the burden at younger ages that compensates for disequalising contributions in old ages. Such changes in mortality rates by age and variations in causes of death has a repercussion on the age pattern of mortality. While there are significant changes in the mortality rates and variations in cause of death over time in the infant, child, and adult age groups; however, the same is modest in middle through old ages which is also evident in terms of modest mortality deceleration at old ages over time^42,63^. As a consequence, the *pattern* of age-specific contributions has remained more or less unchanged and a modest structural change in causes of death is witnessed in India.

On the other hand, developed countries such as Japan, Sweden, Switzerland, Singapore, Australia, Germany, Russia, the USA, and other developed countries show significant changes in the *pattern* of age-specific contributions as well as causes of death structure, and importantly high causes-of-death variation^23,64–66^. Bergeron-Boucher et al.^13^ for low-mortality countries demonstrate significant rise in cause-of-death variation, measured by entropy, since the early 1990s. Yoshinaga and Une^67^ for Japan demonstrate dramatic changes in the cause of death structure from the dominance of tuberculosis and pneumonia until the 1960s to cerebrovascular diseases between 1970 and 1990s, and heart diseases other than ischemic heart disease since 2000s, along with shift in the major contributing age group, i.e. from 0–4 years to 75–84 years. Japan manifests changes in causes of death structure many times and swift changes in the *pattern* of age-specific contributions to gain one of the highest e_0_ in the world. Denmark show a significant change in the contribution of middle age group which was negligible during 1960–75, and increased to large, significant contribution of ~ 50% during 1995–2014^17^. Such mortality changes at middle through old ages^64,67,68^ concomitant of causes of death structure impels a stronger transformation in the distribution of age at death in order to keep epidemiological transition apace in these developed countries^1,69^.

On the contrary, India lacks such demographic developments. Linear increase in e_0_ depicts a smooth progression in mortality transition, however, scrutinization of G_0_ reveals high inequality in age at death caused by NCDs, similar *pattern* of age-specific contributions over time, and modest changes in the cause of death structure mainly attributed to CDs in the studied period. Yadav and Arokiasamy^70^ demonstrate a change in the causes of death structure wherein the burden of NCDs surpassed CDs in mid-1980s; however, thereafter, since the early 1990s the age pattern of mortality of many NCDs marginally changed accounting for toll of deaths (Figs. 5 and S1). The results in this study showed high inequality in mortality distribution causes by many NCDs which was camouflaged in the analysis of e_0_. The outcome of the study demonstrates that high inequality mortality caused by many NCDs have been responsible for slowing down the advances in the epidemiological transition during a period of 30 years between 1990 and 2019. Furthermore, the untimely and behindhand programs for chronic NCDs^71^ has already instigated morbidity expansion that rather strengthens dual burden of diseases in India.

In particular, India lacks a mortality decline at the middle ages. The mortality decline at middle ages dominated by high mortality rates of chronic NCDs presents three major benefits: (a) large equalising effect for decline in G_0_, (b) shift in the threshold age, and (c) benign disequalising contributions in old ages. This demographic development can be achieved by reduction in toll of deaths caused by enteric infections, respiratory infections and tuberculosis, cardiovascular diseases, and chronic respiratory diseases in the middle age group of 50–69 years. It appalling to note that despite a linear increase in e_0_, India shows a modest reduction in premature mortality that in turn undermines the fundamental demographic processes such as mortality compression, the phenomenon of high e_0_ and low G_0_, and morbidity compression. The middle age group provides a compatible possibility for progress of these fundamental demographic processes and epidemiological as well as mortality transition comparable to that in developed countries in a short time. The developed nations demonstrate advances in the third and later phases^6,7^ of epidemiological transition by a greater role of middle age group for structural changes among NCDs, i.e. in the age pattern of mortality, causes of death structure, and importantly causes-of-death variation.

The Ministry of Health and Family Welfare (MoHFW) in its recent health report National Multisectoral Action Plan (NMAP) for Prevention and Control of Common Noncommunicable Diseases (2017–2022)^72^, National Programme for Health Care of Elderly (NPHCE)^73^ and National Programme for Prevention and Control of Cancer, Diabetes, Cardiovascular diseases and Strokes (NPCDCS)^74^ strategizes for the assessment of economic and mortality burden of NCDs by strengthening patient data and harmonization of disease data. Under NPCDCS, the MoHFW has proposed to developed standard protocols for data collection, analysis, and reporting of data for different NCDs services at all levels. For this purpose, the Health Management Information System (HMIS) is also leveraged to identify linkage modalities with NCDs services. The interpretation of the results could be better explained with a harmonized data between other sources of data such as Sample Registration System (SRS), Medical Certification of Causes of death (MCCD), and MoHFW, and other health survey data. However, for India, details of causes of death mapped with ICD classification are not available by age, sex and residence for a long period. Also, the use of detailed GBD data needs cautious interpretations^50,51,75,76^. While the life table estimates between GBD data and SRS are very close; nonetheless, synchronous details of causes of death since the early 1990s are available in GBD data.

The national NCD monitoring framework in way forward has prioritise the reduction of premature mortality from 10 to 25% in another five years in the age group of 30–70 years caused by cardiovascular diseases and chronic respiratory diseases^72^. The outcomes of this study point out that surveillance of population in the middle age group of 50–69 years for NCDs, especially COPD, asthma, and pneumoconiosis, can reduce premature mortality in a short time. Also, screening and diagnosis in a narrow age interval lessens the burden on public health system and ascertain effective utilization of limited sources. The contribution of middle ages to mortality decline has been undermined just because of low threshold age in India. This remained neglected since a long time in the policy frameworks as well as research studies. The MoHFW need to prioritise for the surveillance and investment in the middle age group of 50–69 years which promises high possibility to reduction in the premature mortality.

## Conclusion

The study reveals (1) large, significant contributions of CDs for reshaping the distribution of age at death and (2) an exacerbated predicament offset by the intrusion of NCDs for a high inequality in age at death. During a period of 30 years between 1990–1994 and 2015–2019, the structural changes in causes of death has been attributed to CDs and marginally to NCDs. A subtle contribution of NCDs to the transformation in distribution of age at death is evident.

The progression in epidemiological transition is modulated mainly by two factors: (a) moderate contribution of adult age group and (b) greater mortality decline attributable to CDs. The role of NCDs is emplaced at middle and higher ages and the pace of epidemiological transition has been modest because of low threshold age in India; importantly, the possible contribution of middle age group is constrained attributable to high inequality in NCDs. The study reveals the high inequality in age at death in India caused by NCDs at middle (50–69 years) ages is the priority to deal effectively in policies and programs. These are underlying reasons for the prolonged phenomenon of dual burden of diseases in India. In a long period of 30 years, India has shown modest changes in causes of death structure. It rather manifests a divergence from that of oversimplified the Omran’s epidemiological transition.

Our study outcomes highlight the urgent tuning of policies targeting middle-aged persons as their survival can make a shift in the threshold age, contribute to structural changes in cause of death, and low inequality in age at death. This will lessen the dual burden of diseases in India for a demographic leap, morbidity compression, prevent health losses and increase lifespan spent in good health.

## Supplementary Information

Supplementary Information 1.

Supplementary Information 2.

## Data Availability

The datasets generated and/or analysed during the current study are available in the Global Burden of Disease Study 2019 (GBD 2019), Institute for Health Metrics and Evaluation (IHME), United States, http://ghdx.healthdata.org/gbd-results-tool^46^.

## Abbreviations

GBD: Global burden of diseases
IHME: Institute for health metrics and evaluation
NCDs: Noncommunicable diseases
CDs: Communicable diseases
ASDR: Age-specific death rates
ACSDR: Age-cause-specific death rates
ICD: International classification of diseases
COPD: Chronic obstructive pulmonary disease
IMR: Infant mortality rate
U5MR: Under-five mortality rate
MMR: Maternal mortality ratio
MoHFW: Ministry of health and family welfare
NPCDCS: National programme for prevention and control of cancer, diabetes, cardiovascular diseases and strokes
MCCD: Medical certification of causes of death
SRS: Sample registration system
HMIS: Health management information system
NPHCE: National programme for health care of elderly
NMAP: National multisectoral action plan

## 22 causes of death

CRD: Chronic respiratory diseases
CVDs: Cardiovascular diseases
Dia&k: Diabetes and kidney diseases
DigeD: Digestive diseases
Enter: Enteric (diarrhea and typhoid) infections
HIV/A: HIV/AIDS and STI
Mat: Maternal disorders
Menta: Mental disorders
Muscu: Musculoskeletal disorders
Neo: Neonatal disorders
Neopl: Neoplasms
Neuro: Neurological disorders
NTD&M: Neg. tropical diseases and malaria
NutrD: Nutritional deficiencies
ONCDs: Other noncommunicable diseases
Otinf: Other infectious diseases
RI&T: Respiratory infections and tuberculosis
SelHV: Self-harm and interpersonal violence
SkiSu: Skin and subcutaneous diseases
SubUs: Substance use disorders
Trans: Transport injuries
Unint: Unintentional injuries
