# Supplementary figures and images for: The role of age inequalities in cause of death in the slow pace of epidemiological transition in India
Source: Sci Rep. 2022 Nov 24;12:20291. doi: 10.1038/s41598-022-23599-7 (PMC9700746; doi:10.1038/s41598-022-23599-7)

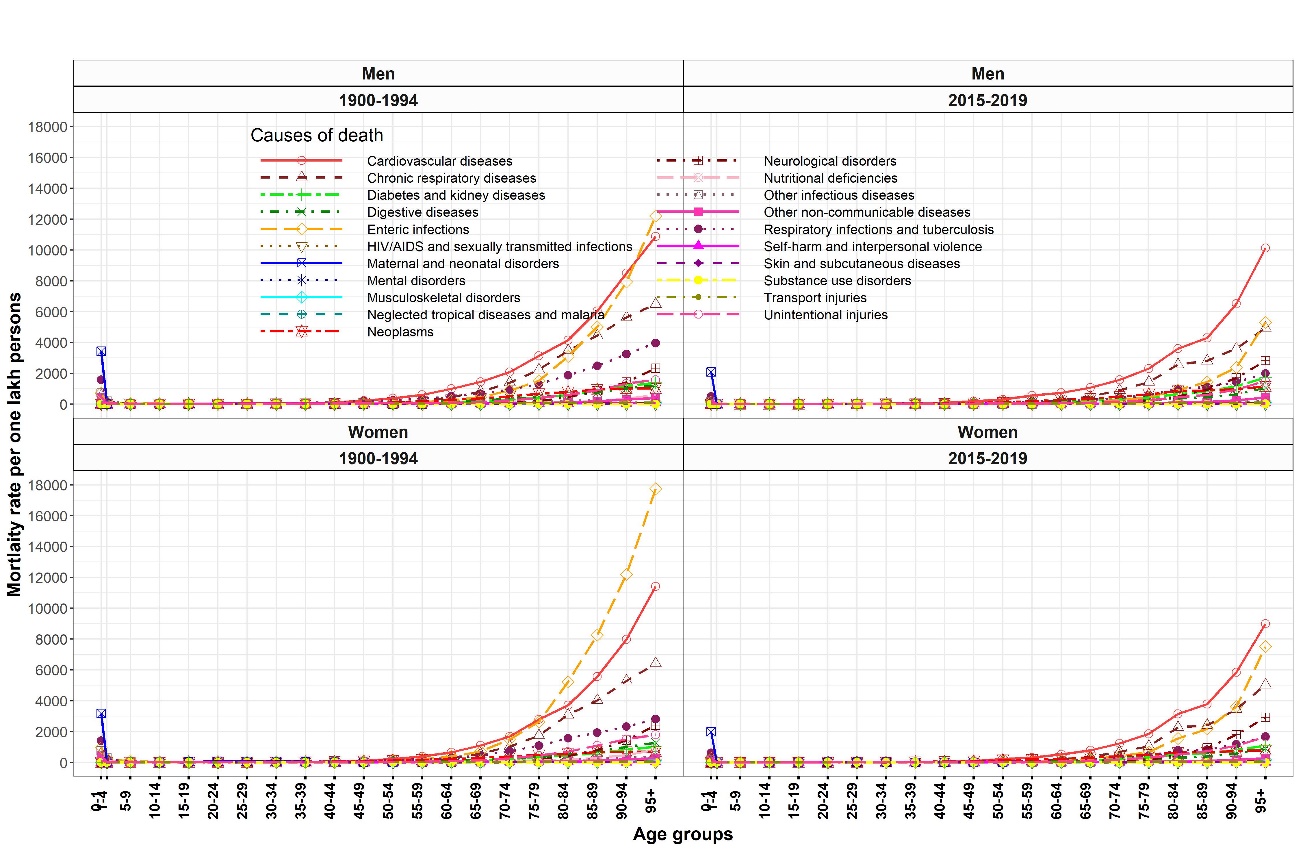


**Supplementary Fig. S1: Age pattern of mortality by causes of death, men and women, India, 1990-1994 and 2015-2019**

Supplement: Supplementary file 1 — Supplementary Information 1. [file 41598_2022_23599_MOESM1_ESM.docx]

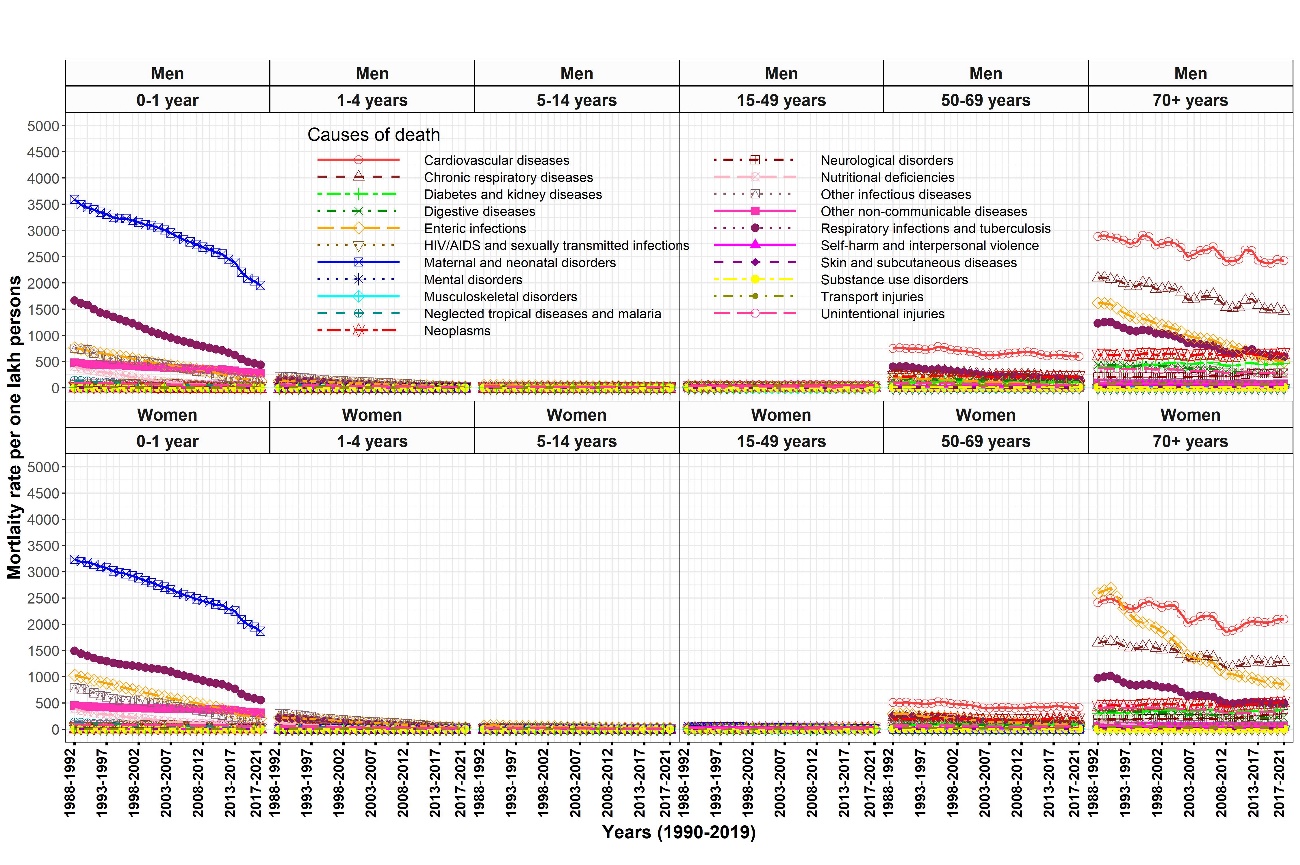


**Supplementary Fig. S2: Trends in the mortality rate of causes of death by age and sex, India, 1990-2019**

Supplement: Supplementary file 2 — Supplementary Information 2. [file 41598_2022_23599_MOESM2_ESM.docx]
